# Development of an Eye Model With a Physiological Blink Mechanism

**DOI:** 10.1167/tvst.8.5.1

**Published:** 2019-09-03

**Authors:** Chau-Min Phan, Hendri Walther, Ha Qiao, Ra Shinde, Lyndo Jones

**Affiliations:** 1 Centre for Ocular Research & Education (CORE), School of Optometry and Vision Science, University of Waterloo, Waterloo, ON, Canada; 2 Manipal Academy of Higher Education, Manipal Institute of Technology, Madhav Nagar, Manipal, Karnataka, India

**Keywords:** in vitro, eye model, blink, polyvinyl alcohol, PVA

## Abstract

**Purpose:**

To develop an eye model with a physiological blink mechanism.

**Methods:**

All parts of the eye model were designed using computer-aided design software. The eyelid consisted of a unique 3D printed structure containing teeth to physically secure a flexible membrane. Both the eyeball and eyelid membrane were synthesized using polyvinyl alcohol (PVA). Four molecular weights of PVA (89–98, 85–124, 130, and 146–186 kDa) were tested at a range of concentrations between 5% and 30% weight/volume. The wettability and water content of these materials were compared with the bovine cornea and sclera. The model was connected to a microfluidic pump, which delivers artificial tear solution (ATS) to the eyelid. A corneal topographer was used to evaluate the tear break-up and tear film regeneration.

**Results:**

The eyelid flexes and slides across the eyeball during each blink, which ensures direct contact between the two surfaces. When loaded with an ATS, this mechanism evenly spreads the solution over the eyeball to generate an artificial tear film. The artificial tear film in this eye model had a tear break-up time (TBUT) of 5.13 ± 0.09 seconds at 1.4 μL/min flow rate, 6 blinks/min, and <25% humidity.

**Conclusions:**

This model simulates a physiological blink actuation and an artificial tear film layer. Future studies will examine variations in flow rates and ATS composition to simulate clinical values of TBUT.

**Translational Relevance:**

The eye model could be used to study in vitro TBUT, tear deposition, and simple drug delivery.

## Introduction

In vivo human studies remain the gold-standard to test new products for their overall performance. However, in vivo studies are extremely expensive and time consuming, and therefore are not suitable to test early stage development of materials, where a rapid “pass-fail” criteria is critical. Another drawback of in vivo testing is that it is difficult to account for the variations between subjects, and therefore isolate the effects of a particular variable. In vitro studies, due to their controlled nature, help fill this gap by providing insights to the “how” and “why” of the underlying mechanisms.^1^ In addition, they also serve as a useful tool for early stage testing and screening of new materials.^1^ Improved, physiologically relevant in vitro eye models would save companies hundreds of thousands of dollars in wasted research and development resources by appropriately vetting early ideas and prioritizing those materials that should go forward for animal or human clinical trials.

Two particularly important tests for ophthalmic biomaterials, relating to comfort, are deposition of tear film (TF) components^2^–^11^ and tear break-up time (TBUT).^12^,^13^ Unfortunately, current in vitro eye models are extremely rudimentary and unable to simulate these complex physiological mechanisms. Most in vitro studies are performed in a simple glass vial, in which the entire material is bathed in a solution of simulated tear solution.^3^,^7^–^11^,^14^,^15^ Factors such as a low tear volume, slow tear flow and replenishment, intermittent air exposure, and the blink actuation, all of which impact tear deposition, are absent from such a simplistic vial-based model. Not surprisingly, results obtained from in vitro studies are often not predictive of in vivo outcomes.

The aforementioned reasons have led various groups to develop in vitro eye models that attempt to mimic key parameters on the eye.^15^–^26^ Our group has previously developed an in vitro model designed to physiologically mimic tear flow, tear volume, air exposure, and the mechanical rubbing produced during blinking.^17^,^18^,^20^–^22^,^27^ Not surprisingly, results obtained for TF deposition on contact lenses using this model revealed stark differences to a simple vial model.^17^,^18^ However, while the model presented an arguably enhanced testing alternative than a vial, it still lacked the physiological blink mechanism that is vital for adequately simulating the dynamic TF layer.

The underlying problem with previous in vitro eye models were that they were made from hydrophobic polymers, such as polydimethylsiloxane.^17^,^18^,^20^–^22^,^27^ Consequently, these materials were unable to simulate two critically important aspects of the corneal surface: wettability and water content (WC). In the eye, wettability is important for the TF to spread over the corneal surface.^12^ The cornea is fairly hydrophilic due to the presence of highly glycosylated transmembrane mucins.^12^,^28^ Additionally, wettability also helps provide the viscous forces and capillary action to hold devices mounted on the eye, such as a contact lens.^29^ However, appropriate WC values would better simulate penetration of molecules, such as drugs, through the cornea.^30^ To address these limitations, a hydrophilic hydrogel material of an appropriate nature that would mimic the wettability and WC of the eye may be a valuable addition to eye models reported to-date.

One material to consider is polyvinyl alcohol (PVA), which has been used previously to synthesize biocompatible materials^31^–^34^ with a high WC.^35^ The properties of the material can be tailored to specific applications by varying molecular weights of the polymer,^36^ cross-linking methods,^31^,^32^,^36^–^38^ solvent composition,^39^,^40^ and incorporation with other polymers and nano-composites.^41^ Studies have shown that PVA-based materials can be designed to be potential substitutes for both artificial cornea^42^ and human vitreous humour.^43^ The polymer is also inexpensive and readily available in comparison to other hydrogel polymers. The purpose of this study was to develop a blink model capable of simulating physiological tear break-up.

## Methods

### Design Software

The designs for the eyeballs, eyelids, and acrylic chambers were designed using a 3D modelling software and a computer-aided design (CAD) program.

### Eyeball

**Figure 1:**
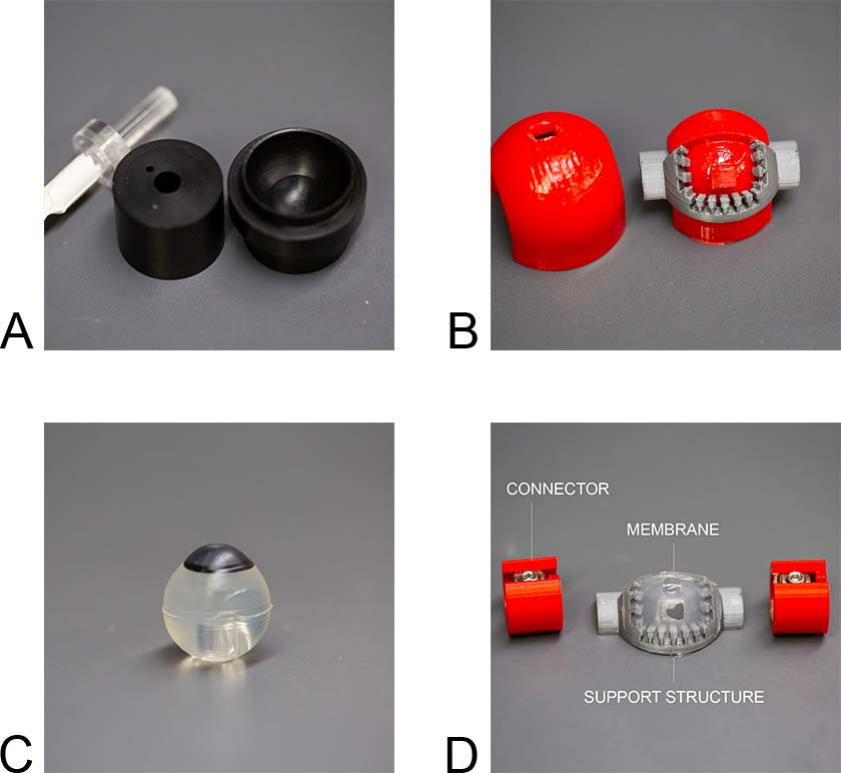
(A) CNC machined molds for the eyeball and (B) 3D printed molds to form the eyelid. (C) Molded eyeball and (D) eyelid from PVA. The eyelid is supported by a 3D printed support structure. A hole in the membrane allows for tear fluid to flow into the eyelid. Two connectors (in red) attach the eyelid to the actuation mechanism.

The molds for the eyeballs were computer numerical control (CNC) machined from acetal plastic to ensure a smooth finish on the corneal surface (Fig. 1A). The eyeball's designed dimensions were 22.5 × 22.5 × 24 mm for height × width × axial length. The molds consisted of two parts, the anterior half to shape the front portion of the eye, and the posterior half to form the back of the eye (Fig. 1). Additionally, the posterior mold has two holes to allow for filling and the escape of any excess polymer solution. A 1/4” diameter acrylic rod is used to create a cylindrical hole cavity inside the eyeball to allow for mounting to the acrylic chamber.

### Eyelid

The molds for the eyelids were 3D printed using polyethylene terephthalate glycol (PETG) on a commercially available 3D printer, TAZ5 (LulzBot, Loveland, CO). The printer uses fused deposition modeling (FDM) to print the parts layer by layer. The print detail was set to high on a 0.35-mm nozzle, with a layer resolution of 0.075 mm. PETG was chosen due to its water resistant properties, but other 3D printer filaments could also be used such as polylactic acid (PLA) or acrylonitrile butadiene styrene (ABS).

The eyelid design, shown in Figure 1B, is composed of three key components: the main support structure, and a top and bottom mold. The role of the support structure (38.0 × 21.4 × 12.1 mm) is to hold and secure a flexible PVA membrane. The two corresponding molds are used to cast a flexible polymer onto the support structure. All components were designed to be reusable, but the main support structure could be replaced with every experiment if necessary. A small hole, created during the molding process, is in the polymer to allow for fluid to flow into the eyelid.

### PVA Preparation

Dimethyl sulfoxide and PVA of four molecular weights of 89 to 98, 85 to 124, 130, and 186 kDa (all 99%+ hydrolyzed) were purchased from Sigma Aldrich (St. Louis, MO). The fabrication procedure was adopted from methods previously proposed by Hyon et al.^40^ and Ma et al.^39^ PVA was added to a mixed solvent of dimethyl sulfoxide and Milli-Q water (8:2) to achieve a range of concentrations between 5% and 30% weight/volume (w/v). The mixture was lightly stirred for 5 minutes, and then heated in an oven at 115 to 120°C for 3 hours. After the heating period, the solution of PVA turned viscous and clear. The hot solution was casted in a mold, and gelled at −30°C for 3 hours. A black plastic disc, used to simulate the iris, was placed in the eyeball during the molding process. The iris is used as a contrasting background, which is required to visualize disruption of the overlying TF in order to accurately measure TBUT. The resulting gels were then thawed at room temperature for 30 minutes and removed from their molds. Formulations of PVA between 5% and 10% w/v require three freeze-thaw cycles to gel properly. The organic solvents within the gels were exchanged with water for 3 days by immersing the gels in 500 mL of Milli-Q water, replaced daily.

### Acrylic Chamber

The acrylic chamber (80.0 × 80.0 × 58.0 mm) was made from individual panels (3.175-mm thickness), and then assembled together using methylene chloride. The panels were cut using a commercial laser cutter (Helix 24 Laser, Epilog Laser, Golden, CO). Cylindrical magnets (diameter = 1.5875 mm; thickness = 3.175 mm) were installed in the top and front panels to allow for an easy access point into the chamber. The sides of the chamber are installed with mounted roller bearings to support the actuation of the eyelid.

### Actuation

The actuation of the system is controlled by a Nema 17 stepper motor (Adafruit Industries, New York, NY) with 200 steps, attached to an Arduino motor shield (Adafruit Industries) and a compatible Arduino board (WEMOS D1 R2 Wifi ESP8266). The code was written using the Arduino App with the available open-source code libraries. The rotation of the eyelid is set to a default rotation at 40 steps forwards and backwards at a speed of 600 rotations per minute (RPM), with a pause of 10 seconds for the interblink period.

### Microfluidic Setup

A commercial syringe pump (PHD ULTRA, Harvard Apparatus, Holliston, MA) delivers simulated tear fluid through a hole at the top of the eyelid through a 1.56”-diameter tubing. The flow rate can be adjusted, but for this study the flow was set to 1.4 μL/min. The blink motion of the eyelid spreads this fluid evenly over the eyeball surface.

### Temperature and Humidity

A DHT22 Arduino humidity and temperature sensor, connected to the Arduino compatible board, was used to monitor the temperature and humidity inside the chamber. The information was displayed on an Arduino LCD display screen module (Geekcreit IIC/I2C 1602).

### Artificial Tear Fluid

The recipe for the artificial tear solution (ATS) has been previously described by our group.^14^ The ATS contains various salts, urea, glucose, mucin, proteins, and lipids.^14^

### Tear Break-Up Assessment

The method for in vitro tear break-up has been previously described by our group.^13^ In brief, the tear break-up and regeneration of the tear layer on the eyeball was assessed using a corneal topographer (K5, Oculus, Wetzlar, Germany). The topographer illuminates the eyeball's surface with a series of placido ring images. The first visible distortion of these rings over time, corresponding to tear break-up, is recorded as the in vitro TBUT. The tear flow rate was set to 1.4 μL/min.

### Wettability

The wettability of the gels were measured using the sessile drop technique and advancing contact angle, which has been previously described.^44^,^45^ In brief, the gels were dabbed gently on lens paper to remove excess solution and then centered onto the Optical Contact Analyzer (DataPhysics Instruments GmbH, Filderstadt, Germany).^45^ A 5-μL drop of high performance liquid chromatography (HPLC) grade water was placed on the curved gel at a rate of 2 μL/s and allowed to settle for 2 to 3 seconds. During this time, a video was taken and saved to the computer hardware. A custom software (SCA 20 software, version 2.04, Build 4) was used to analyze the video and determine the advancing contact angles for material (*n* = 3). The left and right contact angles were averaged to report a single value to obtain the advancing contact angle.

### Water Content

The wet weight (WW) and dried weight (DW) of the gels (*n* = 3) were measured using the Sartorius MA 100H (Göttingen, Germany). The gels were dabbed gently on lens paper to remove excess solution before measuring the WW. The gels were then dried for 48 hours in a 45°C oven, and the dry weight was measured. The WC was calculated using the following formula:

$$\newcommand{\bialpha}{\boldsymbol{\alpha}}\newcommand{\bibeta}{\boldsymbol{\beta}}\newcommand{\bigamma}{\boldsymbol{\gamma}}\newcommand{\bidelta}{\boldsymbol{\delta}}\newcommand{\bivarepsilon}{\boldsymbol{\varepsilon}}\newcommand{\bizeta}{\boldsymbol{\zeta}}\newcommand{\bieta}{\boldsymbol{\eta}}\newcommand{\bitheta}{\boldsymbol{\theta}}\newcommand{\biiota}{\boldsymbol{\iota}}\newcommand{\bikappa}{\boldsymbol{\kappa}}\newcommand{\bilambda}{\boldsymbol{\lambda}}\newcommand{\bimu}{\boldsymbol{\mu}}\newcommand{\binu}{\boldsymbol{\nu}}\newcommand{\bixi}{\boldsymbol{\xi}}\newcommand{\biomicron}{\boldsymbol{\micron}}\newcommand{\bipi}{\boldsymbol{\pi}}\newcommand{\birho}{\boldsymbol{\rho}}\newcommand{\bisigma}{\boldsymbol{\sigma}}\newcommand{\bitau}{\boldsymbol{\tau}}\newcommand{\biupsilon}{\boldsymbol{\upsilon}}\newcommand{\biphi}{\boldsymbol{\phi}}\newcommand{\bichi}{\boldsymbol{\chi}}\newcommand{\bipsi}{\boldsymbol{\psi}}\newcommand{\biomega}{\boldsymbol{\omega}}{\rm{WC\ }}\left( \% \right) = {{\left( {{\rm{WW}} - {\rm{DW}}} \right)} \over {{\rm{WW}}}} \times 100.$$

### Bovine Eye Dissection

The bovine eye is commonly used as an alternative model to the human eye.^46^,^47^ The bovine eyes for this study were donated by an abattoir (Cargill, Guelph, Ontario). The eyes were visually inspected for an intact, nondamaged cornea before dissection. They were dissected fresh on the same day that they were received, based on procedures previously published.^48^ First, the excess outer tissues of the eye were separated from the eye and discarded. The removal of the cornea and sclera began with an incision into the limbus. The tissue was rinsed with phosphate-buffered saline solution. To ensure the samples were fresh, the tissues were tested immediately following the dissection. The wettability and WC of the cornea and sclera were measured (*n* = 5).

### Statistical Analysis

Statistical analysis and graphs were plotted using GraphPad Prism 6 software (GraphPad, La Jolla, CA). All data are reported as mean ± SD for *n* = 3, unless otherwise stated. A two-way analysis of variance was used to determine the difference between percent w/v and molecular weights for WC, and contact angle. Posthoc Tukey multiple comparison tests were used when necessary. In all cases, statistical significance was considered significant for a value of *P* < 0.05.

## Results

After the 3-hour heating step, the PVA turned into a clear viscous solution. The viscosity was not measured, but a higher percent w/v appeared to increase the viscosity. Therefore, it was harder to pour and mold with the higher percent w/v PVA. After the casting step, the resulting gels, consisting of PVA and dimethyl sulfoxide, were transparent. However, after exchanging the organic solvents within the gels with water, the lower concentration formulations of PVA turned translucent.

**Figure 2:**
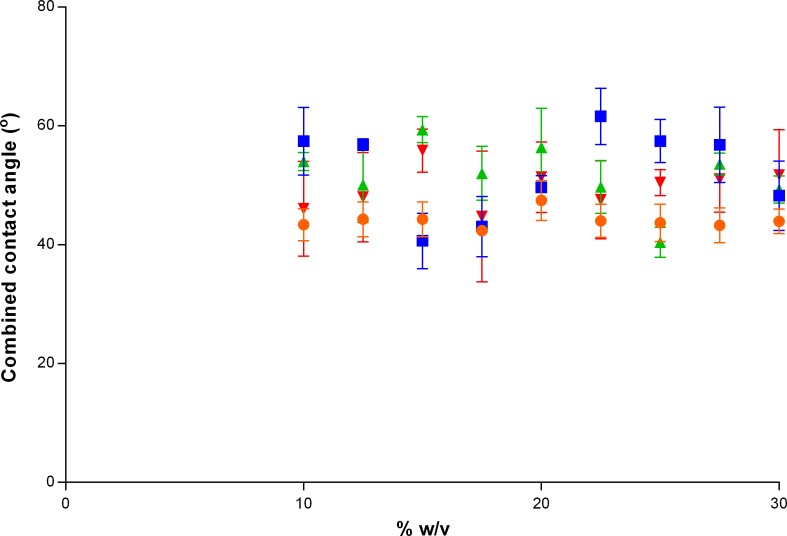
Combined advancing contact angle (°) for different formulations of PVA with molecular weights of 89 to 98 kDa (•), 85 to 124 kDa (▪), 130 kDa (▴), and 146 to 186 kDa (▾) that were tested.

Figure 2 shows the combined advancing contact angle (°) for the different formulations of PVA that were tested. Formulations of PVA less than 10% w/v did not form a stable enough gel to reliably measure the advancing contact angle, so these gels were omitted from the wettability measurements. While there were significant differences in contact angle with different percent w/v (*P* = 0.0008) and molecular weights (*P* < 0.0001), there was no clear trend. The advancing contact angles ranged from 40.4 ± 2.5° (130 kDa, 25% w/v gel) to 61.6 ± 4.7° (85–124 kDa, 22.5% w/v gel).

**Figure 3:**
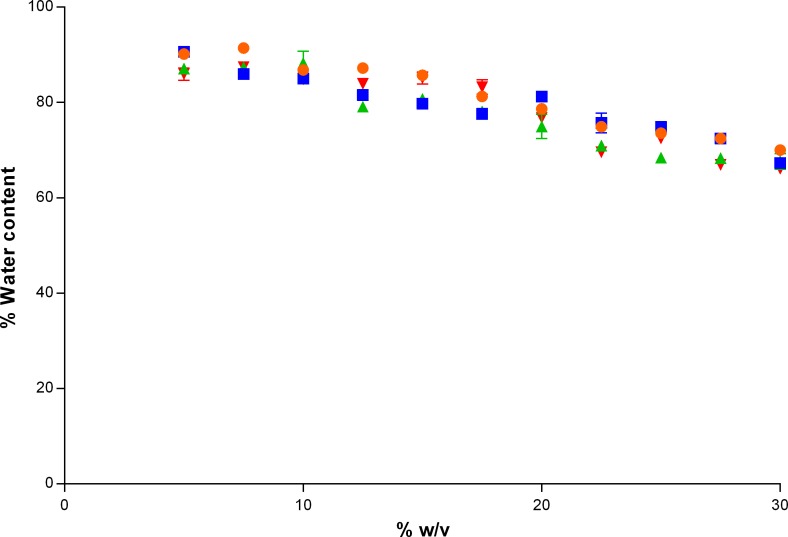
WC (%) for different formulations of PVA with molecular weights of 89 to 98 kDa (•), 85 to 124 kDa (▪), 130 kDa (▴), and 146 to 186 kDa (▾) that were tested.

Figure 3 depicts the WC for the different formulations of PVA that were tested. The WC of the gels decreased with increasing percent w/v (*P* < 0.0001). There were also significant differences in WC between the different molecular weights of PVA used to prepare the gels (*P* < 0.0001), but there was no clear trend on which molecular weights produced the highest or lowest WCs. The WC ranged from 66.1% ± 0.1% (146–186 kDa, 30% w/v) to 91.4% ± 0.2% (89–98 kDa, 7.5% w/v).

The Table reports the contact angle and WC of the cornea and sclera of the bovine eye. These are the target parameters used for comparing the different PVA gels. The 17.5% w/v PVA 89 to 98 kDa formulation was used to synthesize the eyelid and eyeball in this study.

**Table:** Combined Contact Angle and WC of the Cornea, Sclera, Lens, and Vitreous Humor of the Bovine Eye (n = 5)

Part of the Bovine Eye	Contact Angle (°)	WC (%)	Human Eye WC (%)
Cornea	28.0 ± 7.2	80.9 ± 1.2	86^49^
Sclera	56.7 ± 6.5	64.8 ± 0.7	70^50^

**Figure 4:**
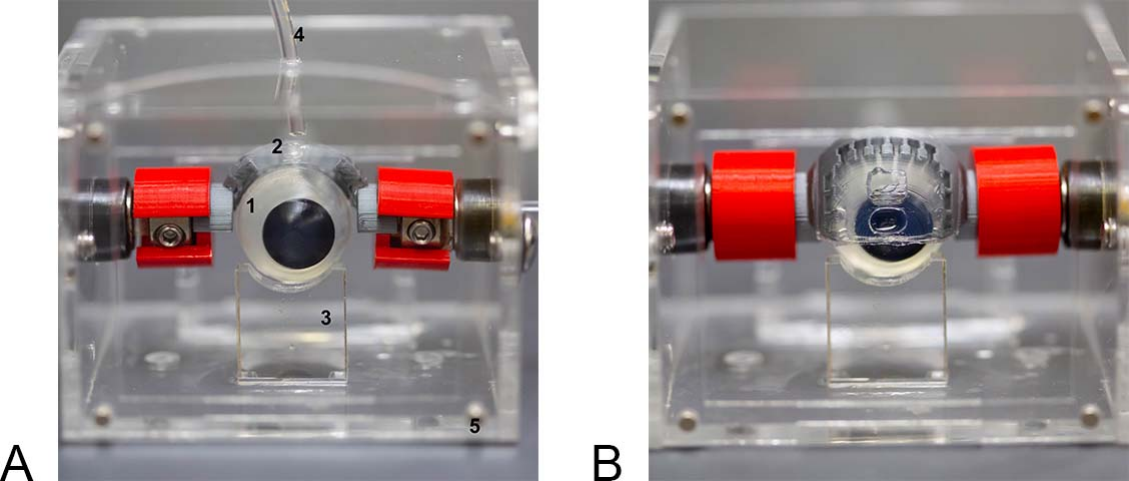
(A) The fully assembled blink model: one eyeball, two eyelids, three lower eyelids, four tubing attached to a microfluidic pump, and five acrylic chambers. (B) Eyelid in the closed position.

Figures 1C and 1D show an example picture of a molded eyeball and eyelid from PVA. The 3D printed support structure for the eyelid has teeth (Fig. 1) to secure the eyelid membrane, which is extremely slippery when hydrated. The eyelid is connected to two connectors that mount the eyelid to the actuation mechanism. Figure 4 shows the entire blink unit enclosed in an acrylic chamber, and an example of the eyelid in the closed position. The motor rotates the eyelid back and forth over the top of the eyeball surface, thereby simulating the physiological blink motion. The eyelid flexes as it travels over the eyeball, and when loaded with a simulated tear fluid, evenly spreads the TF over the eyeball. The eyeball is mounted on an adjustable rod, which is locked into position using a fastener. This allows for adjustments of the eyeball's position to increase or reduce the distance between the eyeball and eyelid. The distance is important for ensuring that the TF spreads evenly, but also not too tight that it will cause the eyelid to eject an ophthalmic device (such as a contact lens) placed on the eyeball during a blink.

**Figure 5:**
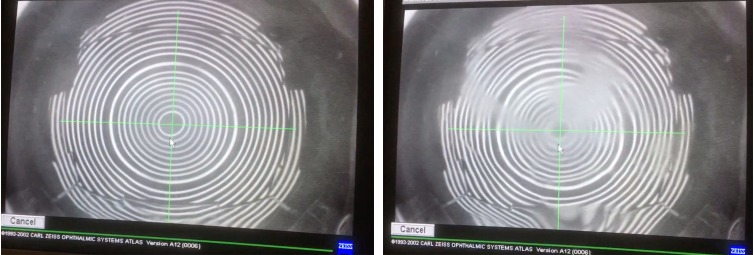
Example of a stable TF using a topographer (left) and tear break-up (right). After each blink, the TF is regenerated.

Figure 5 shows an example of the TF under the topographer. The nondistorted placido rings represent a stable artificial TF. As the eyeball is exposed to the air, the TF begins to break up and the rings become distorted. The average time for tear break-up in this study was 5.13 ± 0.09 seconds (*n* = 3) at a room humidity <25%. The TF layer was effectively and consistently regenerated after each blink.

## Discussion

This study describes a method to construct an eye model that simulates a physiological blink actuation. There were three key technical requirements that needed to be achieved. First, the material properties of the eyelid and eyeball needed to be highly wettable for the TF to appropriately spread. A hydrophobic material such as PDMS, which was used in previous eye models^16^,^17^,^19^–^22^,^27^ would not allow for a proper tear layer to form. Secondly, the eyelid also needed to be flexible to ensure direct contact between the eyelid and the corneal surface. Due to the shape of the front of the eye, which contains two different curvatures for the cornea and sclera, a simple spherical solid eyelid design would result in a large dead space between the eyelid and the cornea. Thirdly, the material for the eyelid also needed to be sturdy in order to withstand thousands of blink cycles during a study period. For example, at a rate of 6 blinks/min, the eyelid would blink 8640 times in a 24-hour study period.

PVA was selected as a base material for the eyeball and eyelid due to its low cost, high WC, mechanical strength, and biocompatibility for future cell studies.^31^–^35^ Furthermore, there are several approaches to cross-link PVA, including chemical and physical cross-linking, which allows for versatility in future material designs.^31^,^32^,^36^–^40^,^51^,^52^ Chemical cross-linking uses cross-linking agents or irradiation to produce permanent gels through covalent bonds.^31^,^51^–^54^ The drawbacks are that toxic reagents and several additional synthesis steps to make a cross-linkable PVA are required.^31^,^51^–^54^ In contrast, physical cross-links are formed through hydrogen bonding, hydrophobic interactions, crystallite formation, and/or entangled chains.^31^,^53^,^54^ We adopted a physical cross-linking approach using a simple freeze-thaw method to synthesize PVA hydrogels (as previously reported by Hyon et al.^40^ and Ma et al.^39^) because of its simplicity and speed.^39^,^40^ Furthermore, because the freeze-thaw method does not require chemicals or a UV curing step, it opened up a wider selection of materials and procedures to mold the gels. One thing to note is that physically cross-linked PVAs are thermo-reversible at higher temperatures.^31^,^53^,^54^ However, this is not a problem for the intended application of the eye model, which will not be subjected to temperatures higher than 40°C. We also noted that for lower concentration formulations of PVA, the gels turned translucent after exchanging the organic solvents within the gels with water. Reduced light transmissibility could be due to phase separation between different hydrophobic and hydrophilic domains within the polymer.^55^,^56^ We hypothesize that as the dimethyl sulfoxide is exchanged for water, the PVA chains in the lower concentration formulations are able to re-arrange in such a manner that creates hydrophobic and hydrophilic regions, resulting in increased phase separation.

The advancing contact angle of the gels tested ranged between 40.4 ± 2.5° and 61.6 ± 4.7°, which suggests that all the gels were highly wettable. While there were significant differences in contact angles between the different gels with regards to percent w/v and molecular weight (*P* < 0.001), there was no obvious trend as to which percent w/v and molecular weight would produce the lowest contact angle. The contact angles measured for the gels were comparable with that of the bovine sclera, which had a contact angle of 56.7 ± 6.5°. The bovine cornea was more wettable, with a contact angle of 28.0 ± 7.2°. These results suggest that the PVA gels synthesized in this study could simulate the wettability of the sclera, and with further modifications, could also mimic the wettability of the cornea. Future studies will attempt to improve the surface wettability by incorporating other hydrophilic components such as mucins, polyethylene glycol, and dextran coatings.^12^,^28^,^57^,^58^

The WC for the gels ranged between 66.1% ± 0.1% and 91.4% ± 0.2%, which were comparable with the WC of the bovine sclera (64.8% ± 0.7%) and the cornea (80.9% ± 1.2%). Overall, the WC of the gels decreased with increasing percent w/v, due to the increase amount of cross-linking.^59^,^60^ The WC measured for the bovine cornea and sclera are slightly less than those previously published for the human eye (see the Table).^49^,^50^

Based on the results of these studies, the 17.5% w/v 89 to 98 kDa gel (contact angle = 43.0 ± 5.1°, WC = 81.3% ± 0.5%) best mimicked the contact angle and WC of the bovine cornea. The 30% w/v 146 to 186 kDa (contact angle = 51.8 ± 7.6°, WC = 66.1% ± 0.1%) closely mimicked the bovine sclera. In this study, the 17.5% w/v PVA 89 to 98 kDa formulation was used to synthesize the eyelid and eyeball. However, we predict that formulations between 15% and 20% w/v PVA for any of molecular weights that were tested would also produce similar results.

One of the biggest design problems encountered during the development phase was mounting a slippery membrane, such as PVA, on an eyelid support structure made from 3D printed material. Our initial attempts included clipping the PVA membrane to a solid eyelid support structure with needles, screws, tape, glue, or tight fitting. While some of these attempts produced better results than others, the end result was that the membrane eventually fell off after several blinks. Finally, an eyelid support structure with teeth structures to secure the eyelid was created and found to be the most efficient design to securely fuse the PVA eyelid in place. Subsequently, the eyelid membrane is molded along with the eyelid support structure to ensure tight binding between the membrane and the support structure.

As the eyelid blinks, a thin artificial TF layer is spread across the corneal surface as seen with the topographer. This artificial TF layer has a TBUT of 5.13 ± 0.09 seconds. This is a relatively fast TBUT, and correlates with those found in patients with dry eye symptoms.^61^ The short TBUT could be attributed to a lower wettability of the artificial eyeball than an actual cornea. The human cornea itself is also not very wettable, but the presence of bound mucins significantly increases its wettability.^12^,^28^ Another reason may be the lack of a lipid layer forming on top the artificial TF. While the recipe for the ATS in this study contains the major components of the natural TF, these components, however, are mixed within the ATS. Consequently, there is no distinctive lipid layer in this model. The absence of a lipid layer has been reported to increase tear evaporation by 4-fold.^62^ Future work will attempt to simulate the lipid layer of the TF to simulate normal physiological TBUTs.

The methods described in this study could also work with other types of hydrogels. Ideally, the hydrogels used in this eye model should be highly wettable, durable, and flexible, particularly if they are to be used as eyelids. For the cornea, the hydrogel does not need to be flexible or as durable, which allows for more material selections. In the past, our laboratory used agar as a polymer for the eyeball to evaluate the growth of microbes on a model eye.^63^ Gelatin, collagen, or their derivatives could potentially be used as a material for the eyeball to grow an overlying layer of corneal epithelial cells to better mimic the ocular surface.^64^

One important concern with this eye model is evaporation. As water is lost from the PVA eyelids and eyeballs, their physical properties will be affected significantly.^53^ For this reason, the acrylic chamber is vital to maintain a stable humidity for the eye model during an experiment. The humidity can be kept relatively high by adding a small source of water in the chamber. In future studies, this chamber could also be used to simulate different types of environmental conditions, like the effects of pollen or pollution on contact lenses.

The eyeball in this model can be horizontally positioned closer or further away from the eyelid as desired. The closer the eyeball is to the eyelid, the higher the pressure exerted by the blink. However, we have not measured these pressures. The pressures and viscoelasticity of the eyelid and cornea have been measured previously using a lid tensiometer^65^ and microindentation method, respectively.^66^ Future studies will aim to adjust the parameters of the blink to match physiological pressures.

**Figure 6:**
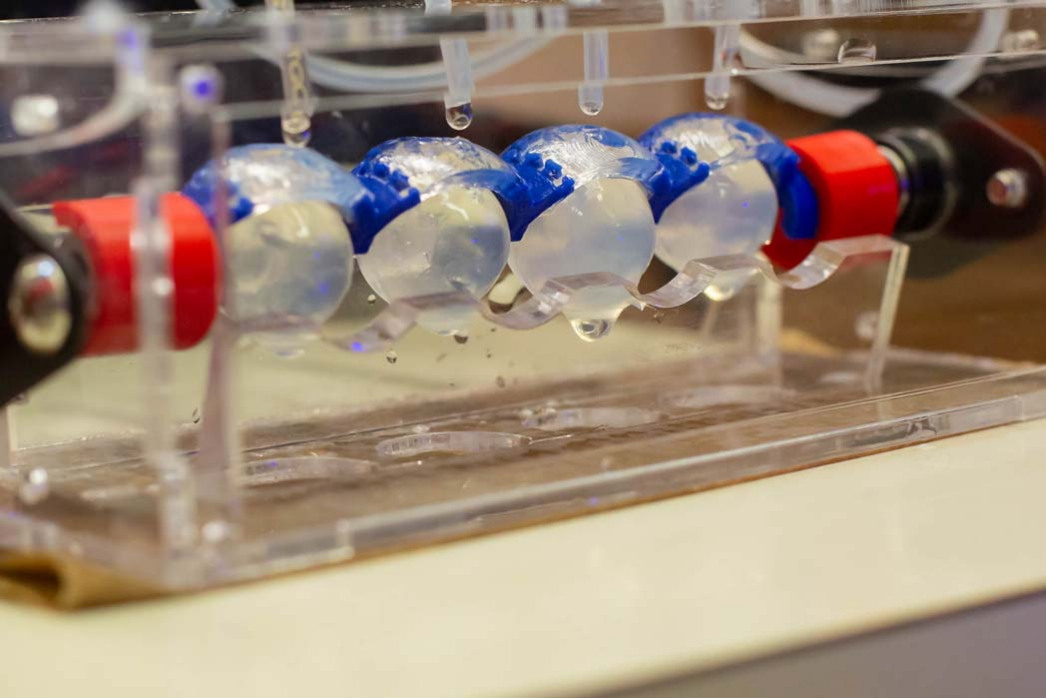
Blink model with four connected eyelids for higher throughput testing.

The model described in this study is for a one-blink unit, but could easily be extended to include several blink units in a chain. Alternatively, the eyelid and chamber can be designed to accommodate a four-unit eyelid as shown in Figure 6. A higher number of simultaneous blink units would be essential for high throughput testing and screening experiments, whereas a one-blink unit would be better for rapid pilot studies.

A microfluidic pump is used to deliver ATS to the eyelid. In this study, a flow rate of 1.4 μL/min was used, which is similar to the average reported physiological tear flow rate for the eye.^67^ However, this flow rate is based on the tears measured on the eye, and may be significantly less than the actual amount of tears initially produced in the glands. We hypothesize that our model eyelid is absorbing a large portion of the tear solution, which consequently reduces the effective flowrate on the eye model. Since tear flow rate would affect TBUTs, as well as deposition of TF components on CLs, determining the correct flow rate is essential. Future studies will therefore use in vivo results to adjust the parameters of this eye model to reach a physiologically relevant state in regards to TBUT and TF deposition.

In conclusion, the eye model developed in this study is capable of simulating a physiological blink mechanism and an artificial TF. The TBUT for this model is slightly lower than clinical results for normal TBUT, which could be due to a lack of a mucin layer, and a distinct lipid layer on the TF. With further adjustment to the parameters, such as tear flow rate, blink speeds, and blink pressure, this model would be a useful tool to study in vitro TBUT, tear deposition, and drug delivery from contact lenses.

## Supplementary Material

Supplement 1
